# A comparative dataset: Bridging COVID-19 and other diseases through epistemonikos and CORD-19 evidence

**DOI:** 10.1016/j.dib.2023.109720

**Published:** 2023-10-24

**Authors:** Andrés Carvallo, Denis Parra, Hans Lobel, Gabriel Rada

**Affiliations:** a Centro Nacional de Inteligencia Artificial, Vicuña Mackenna 4686, Macul, Santiago, Región Metropolitana, Chile; b Pontificia Universidad Católica de Chile, School of Engineering, Department of Computer Science, Vicuña Mackenna 4860, Macul 7820436, Región Metropolitana, Chile; c Pontificia Universidad Católica de Chile, School of Medicine, Avda. Libertador Bernando O'Higgins 340, Santiago 9170464, Región Metropolitana, Chile

**Keywords:** Evidence based medicine, Biomedical text classification, Covid-19, Natural language processing

## Abstract

The COVID-19 pandemic has underlined the need for reliable information for clinical decision-making and public health policies. As such, evidence-based medicine (EBM) is essential in identifying and evaluating scientific documents pertinent to novel diseases, and the accurate classification of biomedical text is integral to this process. Given this context, we introduce a comprehensive, curated dataset composed of COVID-19-related documents.

This dataset includes 20,047 labeled documents that were meticulously classified into five distinct categories: systematic reviews (SR), primary study randomized controlled trials (PS-RCT), primary study non-randomized controlled trials (PS-NRCT), broad synthesis (BS), and excluded (EXC). The documents, labeled by collaborators from the Epistemonikos Foundation, incorporate information such as document type, title, abstract, and metadata, including PubMed id, authors, journal, and publication date.

Uniquely, this dataset has been curated by the Epistemonikos Foundation and is not readily accessible through conventional web-scraping methods, thereby attesting to its distinctive value in this field of research. In addition to this, the dataset also includes a vast evidence repository comprising 427,870 non-COVID-19 documents, also categorized into SR, PS-RCT, PS-NRCT, BS, and EXC. This additional collection can serve as a valuable benchmark for subsequent research. The comprehensive nature of this open-access dataset and its accompanying resources is poised to significantly advance evidence-based medicine and facilitate further research in the domain.

Specifications Table

**Table:** 

Subject	*Health and medical sciences: Public Health and Health Policy*
Specific subject area	*Evidence-based medicine, Bioinformatics*
Type of data	Text Tabular Bin files
How the data were acquired	The dataset consists of 20,047 documents that are related to COVID-19, and they are classified into five types, including Systematic Reviews (SR), Primary Study randomized controlled trials (PS-RCT), Primary Study non-randomized controlled trials (PS-NRCT), Broad Synthesis (BS), and Excluded (EXC). The data was obtained from two sources, the CORD-19 public dataset and Epistemonikos, an online platform where collaborators classify documents into categories and add them to an open-source evidence database. It is worth noting that the data presented in the manuscript are original and produced by the authors. While the data sources are the CORD-19 public dataset and Epistemonikos, one of the authors is the founder of the Epistemonikos foundation and has unique access to the curated dataset, which was not merely scraped from the online platform. We integrated these distinct data sets, adding our proprietary labels to the CORD-19 dataset.The PubMed IDs of the CORD-19 dataset were cross-checked with the Epistemonikos database to ensure the relevance of the data collected to COVID-19. Moreover, a dataset consisting of 427,870 labeled documents that are not related to COVID-19, covering the periods from 2015 to 2019 and 2022 to 2023, is also provided. Trained model weights obtained through training on the above-mentioned datasets are included and can be used as a baseline for future research in this field.
Data format	Raw Filtered Tabular Bin files
Description of data collection	The data collection process for this comprehensive healthcare research dataset commences with a broad search across open-source evidence databases, like the Cochrane Database and Medline. The objective at this stage is to identify potential evidence related to a range of study types. This is followed by a meticulous review, filtering, and labeling stage, undertaken by a dedicated team from the Epistemonikos foundation. They sift through the data, applying explicit inclusion criteria and content analysis to pinpoint relevant evidence. The complete text of the selected articles is examined, enabling the team to label the evidence based on study design. Notably, the data processing stage is attentive to preserving contextual nuances, hence common preprocessing steps like stop word removal or symbol elimination aren't utilized. A two-pronged strategy of cross-checking document metadata and a text-based comparison ensures the effective removal of duplicates. The resultant dataset is a systematic collection of relevant evidence from the Epistemonikos database, providing a robust resource for healthcare decision-makers and researchers. The process culminates in the generation of two distinct datasets, the Epistemonikos Non-COVID dataset, and an enriched CORD19 dataset, achieved by integrating it with the final dataset using filtered PubMed IDs.
Data source location	*Worldwide, PubMed, Epistemonikos, CORD-19*
Data accessibility	Repository name: A Comparative Dataset: Bridging COVID-19 and Other Diseases through Epistemonikos and CORD-19 Evidence. Data identification number: DOI: 8339033 Direct URL to data: https://zenodo.org/record/8339033 Direct URL to GitHub repository: https://github.com/afcarvallo/covid_19_document_type_screening

## 1 Value of the Data


 

• The dataset is valuable for improving the accuracy of machine learning and language models in identifying evidence related to new diseases. By training these models on the COVID-19-related documents and their labels, researchers can develop an automated system that can help in decision-making for treatments and public policies related to emerging diseases. This can significantly speed up the process of identifying relevant evidence and lead to more effective healthcare interventions.
• The dataset can also serve as a starting point for researchers to create more extensive and varied datasets that can be used to train and test new machine learning models. By expanding the dataset to include documents related to other diseases or health conditions, researchers can develop more robust models that can accurately identify relevant evidence across different healthcare contexts.
• The data presented in the manuscript is original and produced by the authors. While the data sources are the CORD-19 public dataset and Epistemonikos, one of the authors is the founder of the Epistemonikos foundation and has unique access to the curated dataset. We integrated these distinct data sets, adding our proprietary labels to the CORD-19 dataset.
• Our dataset, distinct from Cochrane Library data, includes unique labels denoting evidence type, aiding quick identification of key documents such as systematic reviews or randomized primary studies. Sourced from Epistemonikos, our dataset offers users easy access to the most recent and relevant systematic reviews or trials for any disease.

## 2 Objective

The primary aim of this dataset publication is to introduce a systematically organized and categorized collection of 20,047 documents related to COVID-19. This corpus is grouped into five categories, delineated by the nature of their respective studies. The generation of this dataset aims to streamline the advancement of automated systems that bolster evidence-based decision-making in therapeutic choices and public policy. It should be highlighted that the data, while sourced from the CORD-19 public dataset and Epistemonikos, are original and curated by the authors. Notably, one of the authors, a founder of the Epistemonikos foundation, has exclusive access to this curated collection, setting it apart from data scraped from the platform. The integration process involved adding proprietary labels to the CORD-19 dataset, amalgamating these unique data sources. A rigorous cross-validation process was implemented to ensure the relevance of the data sourced from both CORD-19 and Epistemonikos. Alongside this COVID-19 related corpus, we present a dataset comprising 427,870 documents unrelated to COVID-19, retrieved from the Epistemonikos database. The value of this publication lies in its offering of a comprehensive, well-structured dataset. It holds potential to train machine learning algorithms to accurately identify evidence associated with emerging diseases like COVID-19. Our dataset varies from the Cochrane Library data in significant ways. Primarily, our dataset includes labels indicating the type of evidence, which assists users in swiftly identifying the most critical documents, such as systematic reviews or randomized primary studies. Moreover, our data is sourced from the Epistemonikos platform, a unique source of curated data. This curated data is made possible due to the access provided by one of the authors who is the founder of Epistemonikos. Therefore, a user seeking evidence on any disease, for instance, could quickly locate the most recent and relevant systematic reviews or trials in our dataset. Consequently, this dataset provides a beneficial tool for researchers, clinicians, and policymakers, supporting scientifically informed decision-making in the medical field.

### 2.1 Data description

The practice of evidence-based medicine (EBM) relies on scientific evidence to support medical decisions. To facilitate the time-consuming task of finding relevant evidence, health experts categorize documents based on their study type [1,2]. However, with the surge of COVID-19 related research, categorizing a large number of documents has become increasingly challenging [3], [4], [5], [6]. To alleviate this workload, several studies have attempted to use active learning techniques [7,8], which select a subset of articles that are likely to be most informative and representative of the dataset. This work, however, takes a different approach by focusing on the labeling of articles based on their evidence and study design, resulting in a well-organized and labeled dataset.

In evidence-based medicine, document classification is crucial to distinguish the level of relevance of evidence. The types of articles or documents we consider in this dataset are:

- **Systematic Review (SR):** These articles employ a rigorous methodology to identify, appraise, and summarize all evidence relevant to a particular medical issue. They usually comprise Randomized Controlled Trials (RCTs) due to their robustness. Key characteristics include a straightforward research question, a comprehensive and reproducible search strategy, and identification of all relevant studies.
- **Primary Study RCT (PS-RCT):** These studies use a design where subjects are randomly assigned to either an experimental group (receiving the intervention being tested) or a control group (receiving an alternative treatment or placebo). Only articles reporting a randomized trial, trial registries, or protocols are included in this category.
- **Primary Study non-RCT (PS-NRCT):** These studies do not employ a randomized methodology. Instead, they present isolated case results without a robust study design. They can include qualitative or quantitative designs for collecting new data from individuals, populations, or other experimental subjects.
- **Broad Synthesis (BS):** These articles summarize relevant evidence related to a medical issue but are less extensive than systematic reviews. They typically synthesize systematic reviews and, occasionally, primary studies. Criteria for inclusion include an explicit search method and a primary objective to synthesize systematic reviews.
- **Excluded (EXC):** This category includes documents that do not meet the criteria of the previous categories and, thus, are not considered relevant evidence.

Each document type in the dataset is classified according to these categories, allowing users to distinguish the relevance of the evidence more effectively. The dataset contains 20,047 labeled documents related to COVID-19, categorized into SR, PS-RCT, PS-NRCT, BS, and EXC, with PS-RCT and SR being preferred due to their robustness as relevant evidence in EBM [9]. The dataset includes a collection of 427,870 labeled documents unrelated to COVID-19, covering the periods between 2015 to 2019 and 2022 to 2023. This study provides language models (bin files) used on the aforementioned datasets as a baseline for advancing research in this field, with the potential to facilitate the development of automated systems that aid in decision-making for treatments and public policies, ultimately contributing to the field of evidence-based medicine.

One potential real-world application of our dataset is in the arena of policy-making, particularly in relation to the COVID-19 pandemic. For example, when a policy-maker needs to make an informed decision regarding a specific treatment for COVID-19, they could consult our dataset. By searching for the treatment name, they would be able to access the latest evidence from systematic reviews or randomized trials, considered as high-quality evidence in evidence-based medicine, which can then guide the policy decision. In a clinical context, consider a healthcare provider seeking evidence-based guidance for the treatment of lumbago. By searching 'lumbago' in our dataset, the provider can immediately identify and focus on the most recent and highest-quality evidence such as systematic reviews or randomized trials.

In order to provide a comprehensive understanding of the contents and utility of our dataset, we have structured this section into several focused subsections. The Dataset Distribution of Classes, Lengths, and Sources subsection provides details on the varied types of documents, their lengths, and their original sources. The Raw Data Example subsection presents a snapshot of raw data from the dataset to provide a tangible understanding of its structure and contents. Lastly, the “Dataset files description” subsection offers explicit information about the specific files available via the dataset URL. Together, these subsections offer a holistic understanding of the dataset's composition, utility, and potential in advancing the field of evidence-based medicine.

### 2.2 Dataset distribution of classes, lengths, and sources

**Fig. 1:**
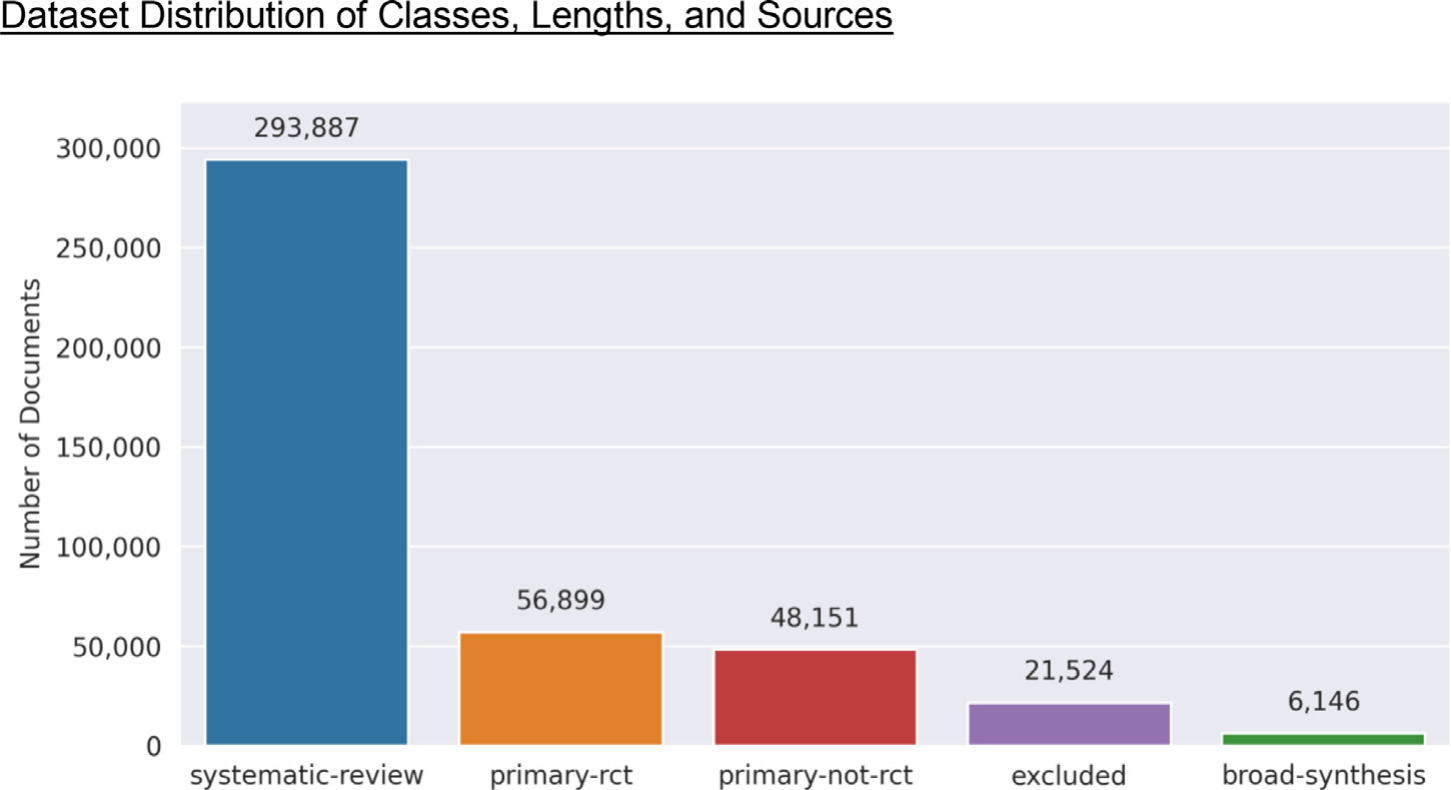
Epistemonikos dataset type of document distribution: This bar chart presents the distribution of different types of articles in the dataset. The x-axis represents the types of articles, while the y-axis shows the count of documents for each type.

**Fig. 2:**
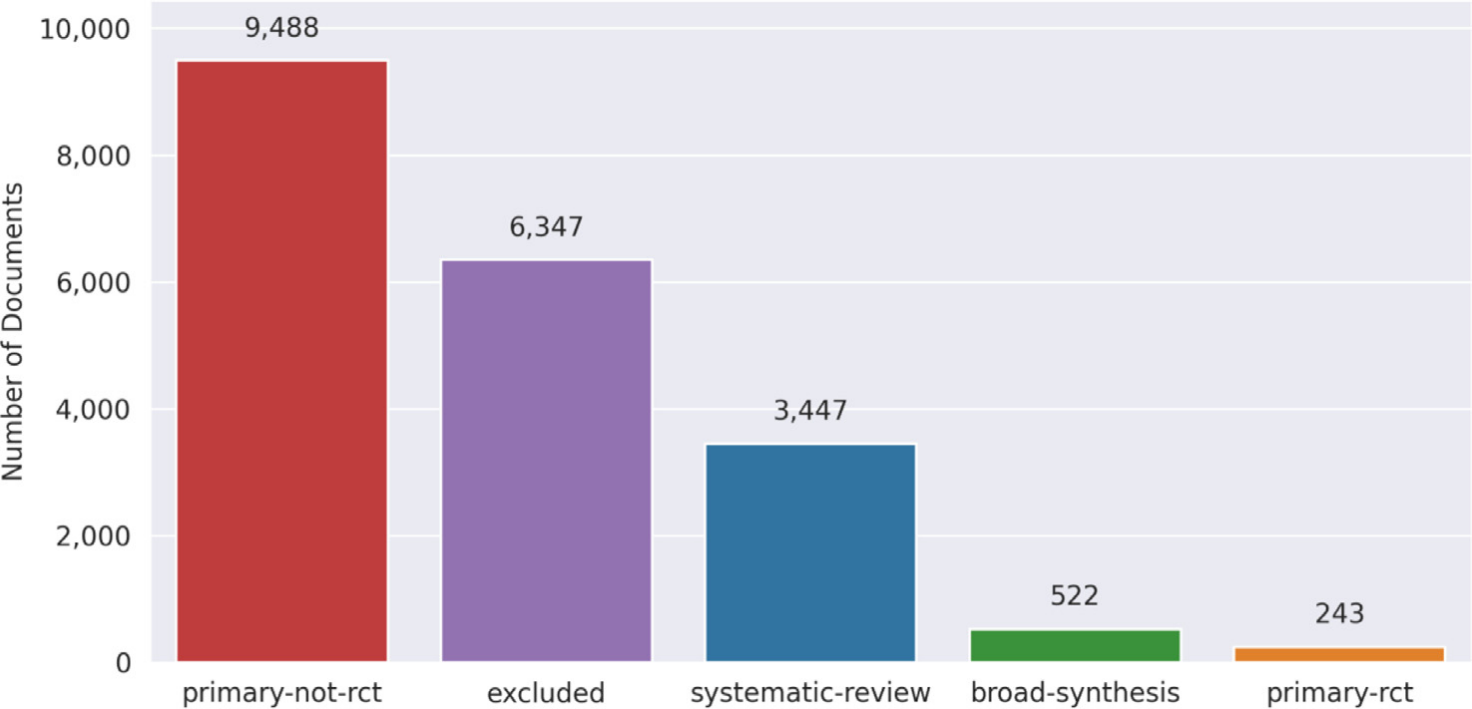
CORD-19 dataset type of document distribution: This bar chart presents the distribution of different types of articles in the dataset. The x-axis represents the types of articles, while the y-axis shows the count of documents for each type.

Fig. 1, Fig. 2 illustrate the distribution of documents across different types within the Epistemonikos and COVID-19 datasets, respectively. In the Epistemonikos dataset, the largest portion of the documents is classified as Systematic Reviews (SR), with a total of 293,887 documents. This is followed by Primary Study Randomized Controlled Trials (PS-RCT) with 56,899 documents, Primary Study Non-Randomized Controlled Trials (PS-NRCT) with 48,151 documents, Excluded (EXC) with 21,524 documents, and Broad Synthesis (BS) with 6,146 documents. On the contrary, the COVID-19 dataset displays a distinct distribution. Here, the most common class is PS-NRCT, comprising 9,488 documents. This is succeeded by EXC with 6,347 documents, SR with 3,447 documents, BS with 522 documents, and PS-RCT with 243 documents. The marked contrast in the distribution of document types between the two datasets could be attributed to the novelty of COVID-19 at the time of data collection. The lack of extensive primary studies on COVID-19 during the initial stages of the pandemic may have limited the availability of systematic reviews, thus impacting the class distribution in the COVID-19 dataset.

**Fig. 3:**
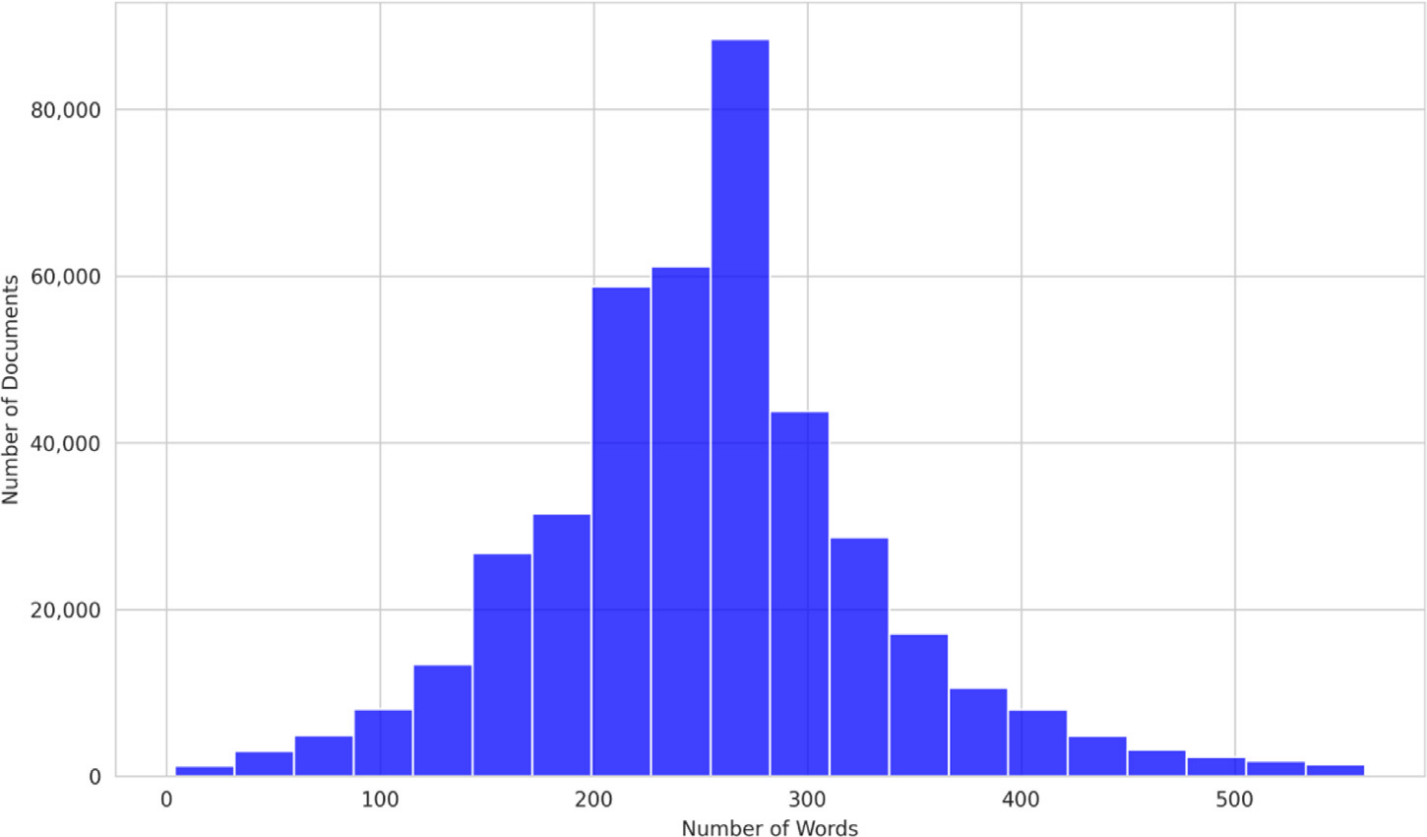
Epistemonikos dataset distribution of document lengths: this histogram illustrates the distribution of document lengths in the corpus. The x-axis represents the length of documents (number of words), and the y-axis shows the number of documents at each length.

**Fig. 4:**
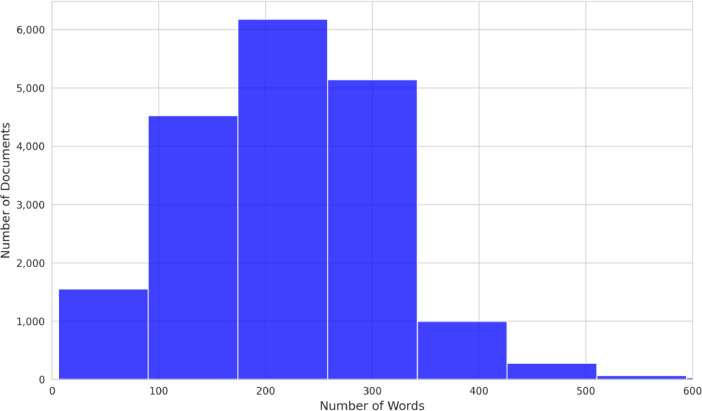
CORD-19 distribution of document lengths: this histogram illustrates the distribution of document lengths in the corpus. The x-axis represents the length of documents (number of words), and the y-axis shows the number of documents at each length.

Fig. 3, Fig. 4 present the distribution of document lengths for the Epistemonikos and COVID-19 datasets, respectively. Both datasets display a similar distribution of document lengths, with the majority of the documents falling within the range of 250 to 300 words. This indicates a broad consistency in the length of abstracts across both the Epistemonikos and COVID-19 datasets, reflecting a general standard in the biomedical literature. The comparability in document lengths between the two datasets provides an even ground for comparative analysis and model generalization.

**Fig. 5:**
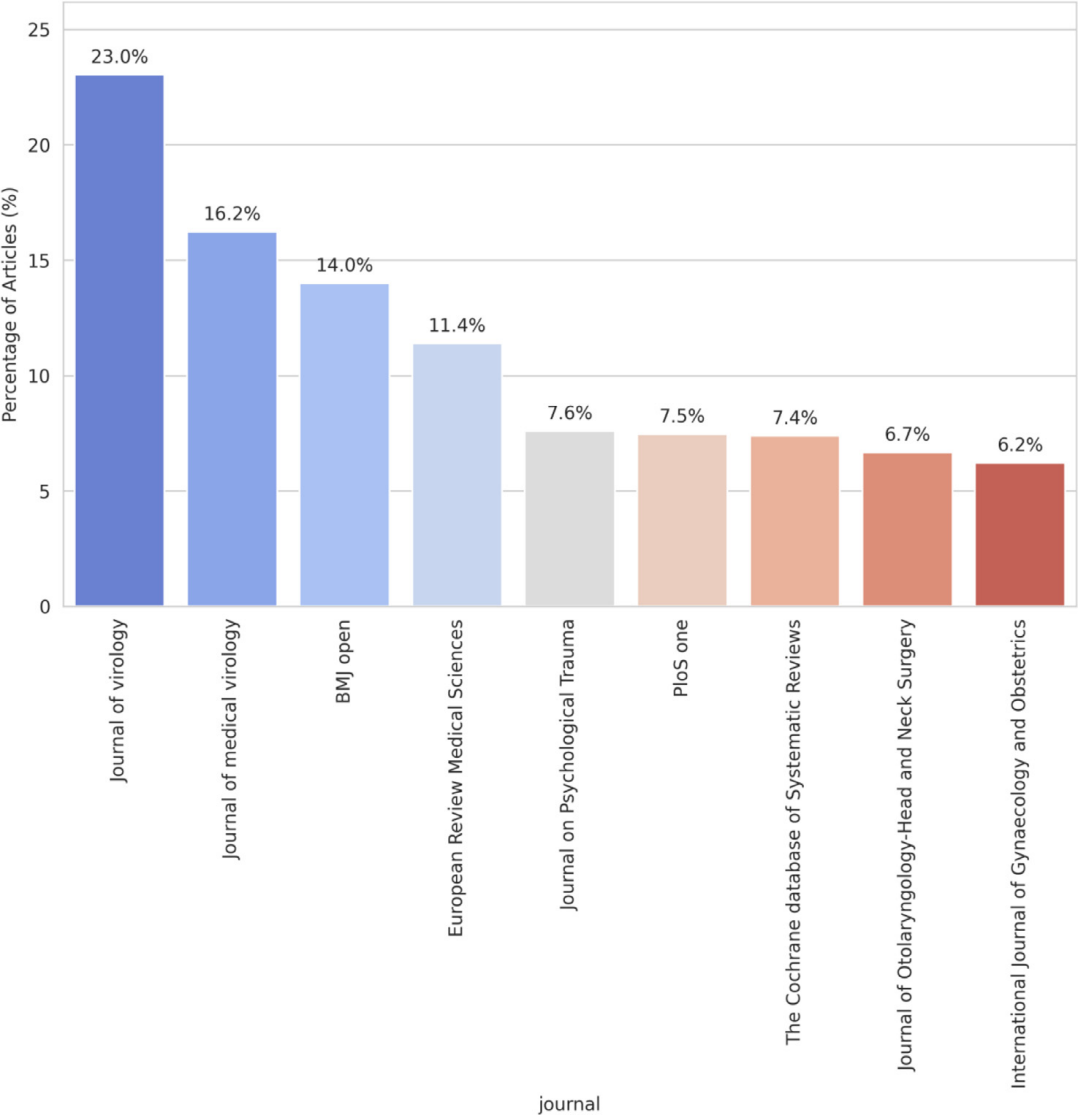
Distribution of the most frequently occurring journals in our dataset. The y-axis represents the percentage of total articles from each journal. The graph provides a visual representation of how different journals contribute to the overall dataset.

As shown in Fig. 5, the articles encompassed within the dataset are predominantly sourced from esteemed academic journals. Foremost among these is the “Journal of Virology,” which constitutes approximately 23% of the dataset's total volume. This is succeeded by the “Journal of Medical Virology,” representing 16.2% of the articles. “BMJ Open” also holds a considerable share, contributing to 14.0% of the dataset. The contributions of the “European Review Medical Sciences” cannot be understated, with its 11.4% representation. In close proximity are the “Journal on Psychological Trauma” and “PloS One,” with respective shares of 7.6% and 7.5%. Additionally, both “The Cochrane Database of Systematic Reviews” and the “Journal of Otolaryngology-Head and Neck Surgery” play pivotal roles, contributing 7.4% and 6.7% to the dataset. Concluding the list is the “International Journal of Gynaecology and Obstetrics,” which represents 6.2% of the dataset. Collectively, Fig. 5 elucidates the comprehensive and varied origins of the articles, emphasizing the richness and diversity inherent in the dataset's scholarly sources.

### 2.3 Region representation of the included article

**Fig. 6:**
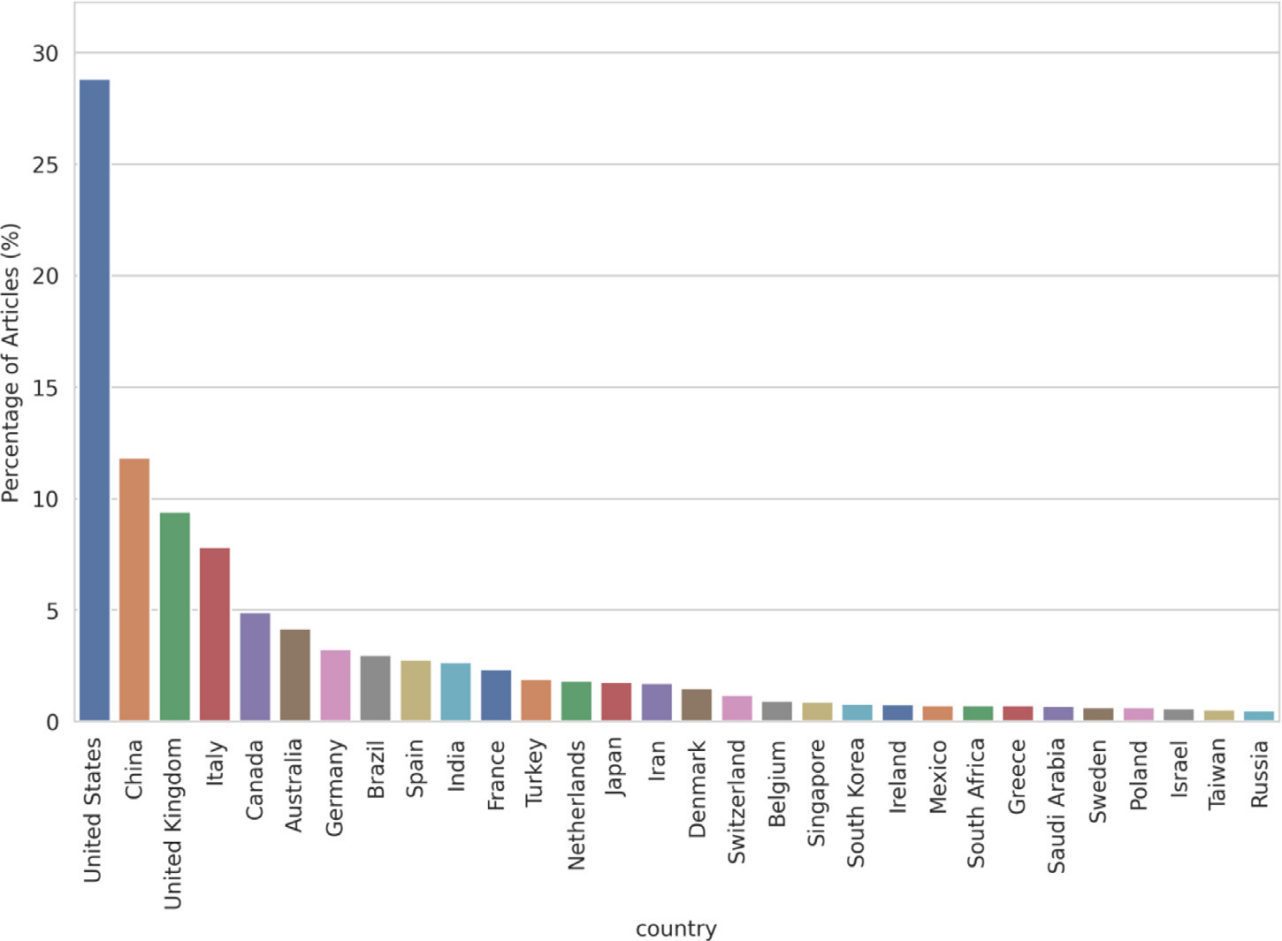
Distribution of scientific publications by country.

Fig. 6 displays a bar chart that depicts the distribution of scientific publications globally. The origin of these publications is determined based on the countries of the institutions with which the authors are affiliated. The data reveals significant disparities between countries in terms of their scientific output. The United States dominates the distribution, accounting for 28.8% of the total publications, almost three times more than the second most publishing country, China, which contributes 11.8% of the total. This substantial gap in output is an important observation in the analysis of global scientific production. The United Kingdom takes the third place with 9.4%, followed closely by Italy at 7.8%. Canada and Australia account for a smaller share of the total publications, contributing 4.9% and 4.2% respectively. Germany (3.2%), Brazil (3.0%), Spain (2.8%), and India (2.7%) also have noteworthy contributions to the total scientific output. France, Turkey, the Netherlands, Japan, Iran, Denmark, Switzerland, and Belgium have proportions ranging from 1.9% to 2.3%. The remaining countries, including Singapore, South Korea, Ireland, Mexico, South Africa, Greece, Saudi Arabia, Sweden, Poland, Israel, Taiwan, and Russia, contribute less than 1% each to the total count of publications. Overall, the bar plot underscores the significant role of countries such as the United States, China, and the United Kingdom in global scientific publication, while also highlighting the substantial contributions from a wide range of other countries. These findings reflect the global nature of scientific research and underline the need for international collaboration in scientific endeavors.

### 2.4 Raw data example

Table 1 presents the structure of the dataset entries. Each row in the table represents an individual document, with details arranged across several columns. The table columns are as follows:

- source: This denotes the database or repository from which the document was retrieved.
- pid: This is the PubMed ID, a unique identifier for the document in the PubMed database.
- title: This represents the title of the document.
- abstract: This provides a brief summary of the document content.
- authors: This lists the authors of the document.
- journal: This column denotes the name of the journal in which the document was published.
- published time: This specifies the date when the document was published.
- url: This column provides a hyperlink to access the full document online.
- label: This denotes the classification of the document based on its type of evidence such as excluded, systematic-review, etc.

**Table 1:** This table outlines the structure of individual entries within our dataset. Each row represents a unique article, with detailed information provided across various fields, such as source, Pubmed ID (pid), title, abstract, authors, journal, published time, URL, and label.

source	pid	title	abstract	authors	journal	published time	url	label
Medline	33165621	Effect of Hydroxychloroquine on Clinical Status ...	Importance Data on the efficacy of hydroxychlo …	Self, Wesley H; Semler, Matthew W; Leither	JAMA	2020-11-09	https://doi.org/10.1001/jama.2020.22240	primary-rct
Medline	33004394	Nutrition screening and assessment tools for …	INTRODUCTION Nutritional challenges are common …	Kristensen, Marianne Boll; Wessel, Irene	BMJ open	2020-10-01	https://doi.org/10.1136/bmjopen-2020-037844	systematic-review

Overall, Table 1 provides a detailed breakdown of each document, including bibliographic details and the assigned evidence type label.

Table 2 shows representative examples for each type of article. A Systematic Review (SR) such as “Efficacy of Antitoxin Therapy in Treating Patients With Foodborne Botulism” examines a broad range of studies on a specific topic, performing a meta-analysis to assess overall effectiveness. In contrast, Primary Studies with Randomized Controlled Trials (PS-RCT) like “Intermittent pneumatic compression to prevent venous thromboembolism” deal with a well-defined intervention and control group. These typically involve large numbers of patients and study the impact of an intervention. Primary Studies Non-Randomized Controlled Trials (PS-NRCT), demonstrated by “The Role of Convalescent Plasma and Tocilizumab in the Management of COVID-19 Infection,” observe patient behaviors under certain conditions. They are cohort studies and may offer comparative analyses, but do not randomly assign patients to intervention or control groups, therefore having a lower level of evidence. Broad Synthesis (BS) as represented by “The relationship between a trusted adult and adolescent outcomes” provides an overview of a particular topic. However, these are not as exhaustive as SR and typically don't involve meta-analysis, but aim to collate and map the existing evidence. Excluded (EXC) articles, such as “Rats, cities, people, and pathogens,” are those that don't fit into the aforementioned categories, including animal studies or ones with topics that do not directly impact human health practices or policies.

**Table 2:** Examples of articles for each type within the dataset. This table showcases representative articles from each category, namely systematic reviews (SR), non-randomized controlled trials of primary studies (PS-NRCT), randomized controlled trials of primary studies (PS-RCT), broad synthesis (BS), and excluded articles (EXC). For each article type, the table provides the title and abstract, to demonstrate the key features and content typical of that category.

Type of article	Title	Abstract
Systematic Review (SR)	Efficacy of Antitoxin Therapy in Treating Patients With Foodborne Botulism: A Systematic Review …	Background Botulism is a rare, potentially severe illness, often fatal if not appropriately treated. Data on treatment are sparse. We systematically evaluated the literature on botulinum antitoxin and other treatments.…
Primary Study RCT (PS-RCT)	Intermittent pneumatic compression to prevent venous thromboembolism in patients with high risk of bleeding hospitalized in intensive care units …	PURPOSE: Venous thromboembolism (VTE) is a frequent and serious problem in intensive care units (ICU). Anticoagulant treatments have demonstrated their efficacy in preventing VTE…
Primary Study non-RCT (PS-NRCT)	The Role of Convalescent Plasma and Tocilizumab in the Management of COVID-19 Infection …	AIM As Coronavirus Disease-2019 (COVID-19) pandemic continues to evolve, the search for safe and effective therapeutic interventions remain essential…
Broad Synthesis (BS)	The relationship between a trusted adult and adolescent outcomes: a protocol of a scoping review.	In this review, we aim to explore the impact and significance of trusted adults on the health and education of adolescents…
Excluded (EXC)	Rats, cities, people, and pathogens: a systematic review …	Urban Norway and black rats are the source of a number of pathogens responsible for significant human morbidity and mortality in cities around the world...

### 2.5 Dataset files description

The dataset provided comprises the following files that can be found in the dataset URL, each catering to distinct aspects of our research:

- Epistemonikos_dataset.tsv: Originating from the Epistemonikos database, this dataset contains attributes such as source, pid, title, abstract, authors, journal, publication date, url, and their classification according to their type of evidence, helping to ascertain their relevance. The dataset includes a collection of 427,870 labeled documents unrelated to COVID-19, covering the years 2015 to 2019 and 2022 to 2023.
- CORD19_full_labels.csv: This file is an adaptation of the original CORD-19 dataset, tailored to our specific domain of Evidence-Based Medicine. The articles contain attributes such as source, pid, title, abstract, authors, journal, publication date, url, and their classification according to their type of evidence, helping to ascertain their relevance. This dataset consists of a categorized collection of 20,047 documents related to COVID-19.
- xlnet.dat: This file contains the weights of the XLNet language model fine-tuned on the Epistemonikos dataset. The primary goal of employing this model was to examine its potential in identifying relevant evidence within COVID-19 articles, in the context of the adapted CORD-19 dataset. The results obtained from this language model can be found in the work done by Carvallo et al [9].
- biobert_epistemonikos_finetuned.dat: Similar to xlnet.dat, this file holds the weights of the BioBERT language model fine-tuned with the Epistemonikos dataset. The purpose of this model was to test its capability to recognize pertinent evidence in COVID-19 articles, again using the adapted CORD-19 dataset for evaluation. The results obtained from this language model can be found in the work done by Carvallo et al [9].

## 3 Experimental Design, Materials and Methods

The objective of this study was to systematically collect, curate, and annotate a dataset of relevant evidence for healthcare decision-making purposes. The data collection protocol was based on Evidence-Based Medicine (EBM) strategies [10] and involved a series of specific steps.

**Fig. 7:**
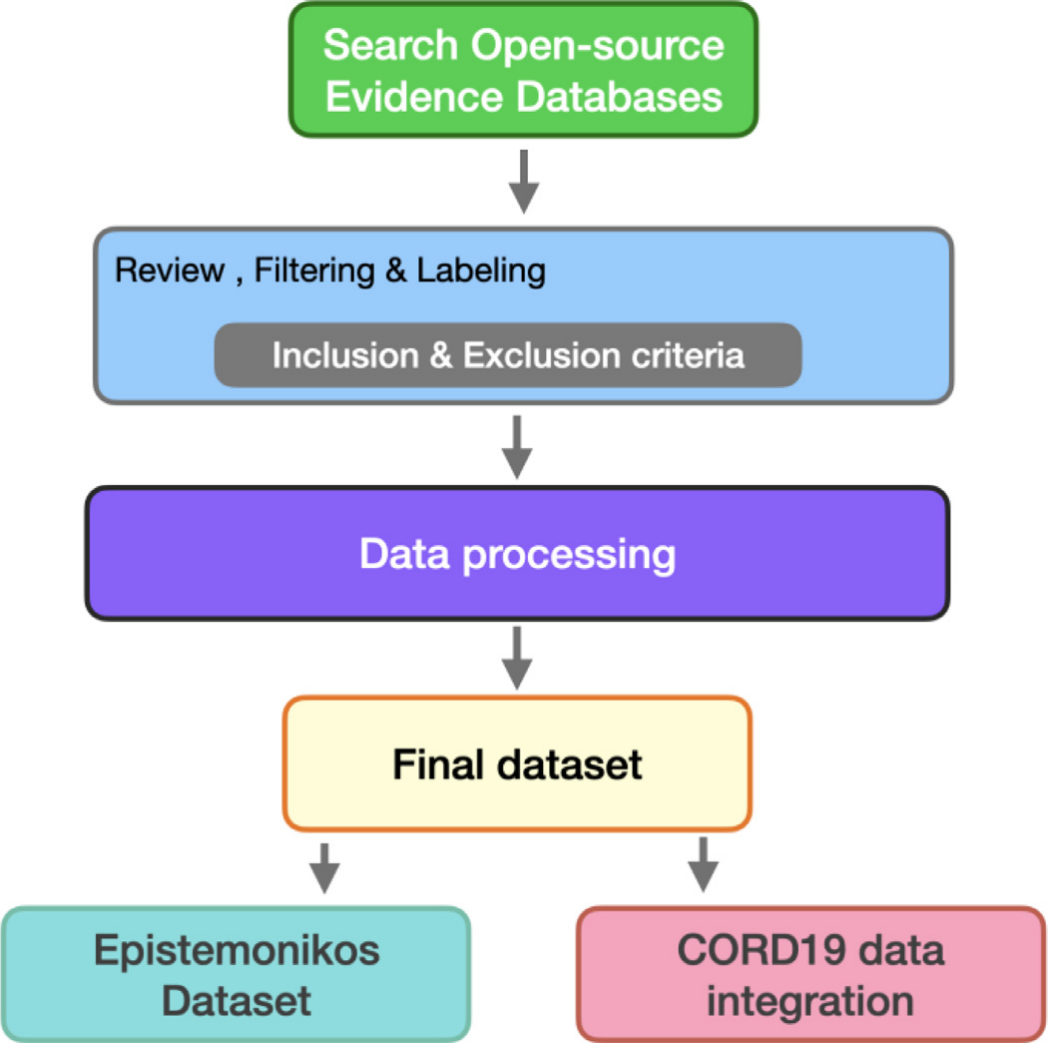
This diagram represents the five stages involved in curating a comprehensive healthcare research dataset: (1) Searching open-source databases for potential studies, (2) Reviewing, filtering, and labeling articles by trained human reviewers, (3) Data processing data (4) Assembling the final curated dataset from Epistemonikos, and (5) Generating two distinct datasets, the Epistemonikos and enriched CORD19 datasets.

As shown in Fig. 7, the process of creating a comprehensive healthcare research dataset is divided into five key stages:

1. Search Open-Source Evidence Databases: The first stage of the process involves searching across various open-source evidence databases such as the Cochrane Database and Medline. The goal here is to identify potential evidence that matches various study types.
2. Review, Filtering & Labeling, Including Exclusion or Inclusion Criteria: The second stage shown in Fig. 6 represents a complex multi-part process. First, a team of about 100 human reviewers affiliated with the Epistemonikos foundation conducts an initial review of the documents, relying on explicit inclusion criteria and content analysis to detect relevant evidence based on relevant evidence based practice questions stated by Davies et al [11]. This stage involves a comprehensive review of the complete text of the chosen articles to identify their associated studies and assign labels based on study design (SR, PS-NRCT, PS-RCT, BS, or EXC). Depending on the type of article, including BS, SR, PS-RCT, PS-NRCT, and EXC, the protocol varied. For example, for BS and SR, we considered articles that explicitly described their method for synthesizing reviews and primary studies and that used at least one electronic database. For PS-RCT and PS-NRCT, we included any type of research study that involved the collection of data from individuals or groups.
  Documents were excluded (EXC) if they did not address a health problem, did not include patient-important outcomes or accepted surrogate outcomes, explored methodological issues that could not be transferred into practice or policy, or did not evaluate individuals or groups of individuals (with exceptions for some topics, such as bacterial resistance to antibiotics or levels of environmental chemicals in food).
3. Data Processing: we do not apply common preprocessing steps such as stop words removal or symbol elimination, as these elements can contribute to contextual nuances in language model training. Regarding duplicate removal, we implemented a two-step strategy. First, we cross-check document metadata for exact matches. Subsequently, we conduct a text-based comparison of the content within each document to detect overlaps.
4. Final Dataset: The fourth stage illustrates the assembly of the final dataset, a comprehensive and systematic collection of relevant evidence directly obtained from the Epistemonikos database. This curated and cleaned dataset represents a valuable resource for healthcare decision-makers and researchers aiming to conduct evidence-based research.
5. Obtention of Epistemonikos and CORD19 datasets: The final stage in Fig. 6 highlights the derivation of two distinct datasets. First, the Epistemonikos Non-COVID dataset is generated by applying filters to the final dataset to exclude any COVID-related evidence. Second, the CORD19 dataset is enriched by integrating it with the final dataset using filtered PubMed IDs. This step enhances the CORD19 dataset by adding more information in the form of article types labels, providing a more comprehensive resource for researchers.

The datasets resulting from this process constitute a crucial asset for healthcare decision-makers and scholars aiming to engage in evidence-based research. These collections of data offer a robust and systematic assemblage of germane evidence, intended to inform and enhance clinical decision-making processes, policy formulation, and the conduct of further research investigations. The data in these collections are derived directly from the Epistemonikos database, and have undergone meticulous curation and cleaning. This ensures that the information is not only proprietary but is also of high integrity and relevance, offering valuable insights and guidance for the healthcare field.

## Ethics Statements

The data have no personal information or institutional references that may compromise the privacy of any parties; therefore, no ethical implications should be declared.

## CRediT authorship contribution statement

**Andrés Carvallo:** Writing – original draft, Validation, Investigation, Methodology. **Denis Parra:** Conceptualization, Methodology, Supervision. **Hans Lobel:** Conceptualization, Methodology, Supervision. **Gabriel Rada:** Data curation, Validation, Resources, Supervision.

## Data Availability

A Comparative Dataset: Bridging COVID-19 and Other Diseases through Epistemonikos and CORD-19 Evidence. (Original data) (Zenodo) A Comparative Dataset: Bridging COVID-19 and Other Diseases through Epistemonikos and CORD-19 Evidence. (Original data) (zenodo) A Comparative Dataset: Bridging COVID-19 and Other Diseases through Epistemonikos and CORD-19 Evidence (Original data) (zenodo)
